# The source of financial contagion and spillovers: An evaluation of the covid-19 pandemic and the global financial crisis

**DOI:** 10.1371/journal.pone.0261835

**Published:** 2022-01-14

**Authors:** Samet Gunay, Gokberk Can

**Affiliations:** 1 Finance Department, College of Business Administration, American University of the Middle East, Egaila, Kuwait; 2 Accounting Department, College of Business Administration, American University of the Middle East, Egaila, Kuwait; University of Almeria, SPAIN

## Abstract

This study investigates the reaction of stock markets to the Covid-19 pandemic and the Global Financial Crisis of 2008 (GFC) and compares their influence in terms of risk exposures. The empirical investigation is conducted using the modified ICSS test, DCC-GARCH, and Diebold-Yilmaz connectedness analysis to examine financial contagion and volatility spillovers. To further reveal the impact of these two crises, the statistical features of tranquil and crisis periods under different time intervals are also compared. The test results show that although the outbreak’s origin was in China, the US stock market is the source of financial contagion and volatility spillovers during the pandemic, just as it was during the GFC. The propagation of shocks is considerably higher between developed economies compared to emerging markets. Additionally, the results show that the COVID-19 pandemic induced a more severe contagious effect and risk transmission than the GFC. The study provides an extensive examination of the COVID-19 pandemic and the GFC in terms of financial contagion and volatility spillovers. The results suggest the presence of strong co-movements of world stock markets with the US equity market, especially in periods of financial turmoil.

## 1. Introduction

The world has faced many viral outbreaks recently; the SARS-COV in 2003, MERS-COV in 2012 and Ebola in 2014. However, none of these outbreaks impacted the world as COVID-19 has. Initial reports of an outbreak started at the end of December 2020 in Wuhan, China, and The World Health Organization (WHO) declared COVID-19 as a pandemic on March 11, 2020. After that declaration, countries started implementing different measures to mitigate the spread of the virus, which had many social and economic effects. Although COVID-19 began as a viral outbreak, it also created a financial contagion in global markets. Due to the slowdown in the global economy, the West Texas Intermediate crude oil price declined to -$37. The International Monetary Fund’s (IMF) World Economic Outlook (WEO) for 2020 forecasted global growth of -3.0% in April, -4.9% in June, and -4.4% in October [1].

In this study, we analyze the impact of the COVID-19 pandemic and the Global Financial Crisis (GFC) on stock markets from the perspective of financial contagion and compare the extent of exposures. The role of equity markets in price discovery and determination of true share values make them crucial platforms for all countries. Additionally, equity markets are essential barometers for the economies as stock prices can incorporate information arrivals and expectations of various market participants. Determinants such as economic development, political stability, and shareholder protection affect the funds that will be shifted in the stock markets. Economic conditions may force individual and institutional investors to liquidate their stock holdings and deposit in other financial instruments. Therefore, the stock market and economic growth have a mutual relationship regardless of the country’s development level because stock markets serve as a means of foreign direct investment (FDI) flow [2]. A well-developed financial system, shareholder protection, and high public governance quality facilitates FDI inflow, reduces the cost of raising capital, and increases per capita economic growth [3]. On the other hand, the integration of countries through the financial linkages and trade channels makes them more vulnerable to the shocks that stem from equity market crashes. For instance, in both the GFC and COVID-19 pandemic periods, it was observed that cross-market linkages had increased the contagious effects and propagation of the crises. Financial contagion sounds like an undesirable situation, as it allows for the transmission of shocks and crises; however, it also allows for the transfers of development across two or more interconnected countries when markets regain investor trust [4]. Therefore, the investigation of externality and financial contagion in tranquil and crisis periods might offer insight for policymakers who oversee these markets and administer necessary regulations and actions.

As [5] discussed, in addition to asset returns, volatility also impacts fundamental components of a company, such as financial position, profit, loss, and cash flows. Additionally, volatility in stock markets is an essential element in investment decisions, and also forces market players to redesign their portfolios and hedging strategies in order to reduce the risks exposed [6]. Thus, wild fluctuations in asset prices are in the interest of various market participants domestically or overseas. As pointed out by [7], fluctuations in equity markets, above the investor decisions, might also have an impact on national economies by changing consumption decisions. As well, [8] show that increased technological developments and policy deregulations enhance the integration of national equity markets with regional and global counterparts. These cross-market linkages may play an essential role in the transmission of these shocks to other countries. For example, the GFC experience and subsequent events showed that these networks increase the speed and effect of financial contagion on interconnected markets. [9] state that economic (e.g., inflation, employment) and financial (e.g., indices, interest rates) news from the US affects other foreign markets. On the other hand, as stated by [10], we may expect to see a lower correlation between emerging and developed countries regarding the co-movements of asset prices; thus, the negative effects of financial contagion and volatility spillovers might be limited in this group of economies. In essence, this situation was already experienced during the GFC. Countries which do not accommodate complex credit derivatives such as credit defaults swaps (CDS) and collateralized debt obligations (CDO) in their economies exhibited lower negative externality to the shocks that originated from the US economy.

All these facts necessitate taking corresponding actions and measures in developed and emerging economies against risks stemming from various financial, economic, or healthcare events. To mitigate these risks, finance scholars, professionals, and policymakers use a variety of measures as a proxy to weigh the foreshocks and estimate the mainshocks. For example, although the COVID-19 pandemic started as an outbreak, the lockdowns, curfews and social distancing measures turned it into a global event in a short period. Still, to reach the pre-COVID-19 economic activity, countries are taking national measures, such as economic stimulus packages and vaccination programs. Some countries have not successfully applied measures and reduced the spread due to conditions such as human mobility and insufficiency in economic power.

These facts observed during the pandemic differentiate itself from the GFC in terms of its basis and essence. However, although their origin and dynamics are different, the impacts of these two crises on financial markets are comparable due to the similarities in financial contagion and spillovers. The study offers an extensive examination of the COVID-19 pandemic versus the GFC regarding financial contagion and volatility spillovers by providing evidence from both developed and emerging economies. Regarding the solidity of the findings, the statistical features of tranquil and crisis periods are also compared under different time intervals for both crises. In selecting the variables, the countries are grouped as emerging or developed markets based on the classification of [11] and [12]. For developed economies, we choose the US, the UK, Italy, and Spain. From emerging markets, we utilize China and Turkey. The US and the UK are the countries that have the highest weights in the MSCI World Index. We selected Italy and Spain as countries that come after the UK, France, Germany, Switzerland, and the Netherlands in the country weights ranking of the MSCI Europe Index. By doing so, evidence is provided for the top and bottom of developed economies.

The following sections of the study present notable literature reviews, explain the theoretical background of utilized econometric models, and finally, summarize this study’s major findings and implications.

## 2. Literature review

Stock markets have great importance for a healthy domestic and global economy. For instance, [13] report the cointegrating relationship between capital market development and economic growth in the long run. Using 12 emerging countries and US data, [9] find that news about economic growth and unemployment of the US impacts emerging stock markets and causes asymmetric volatility. The authors show that positive news affects more countries than negative news. Additionally, [7] documents that monetary policy decisions have a bi-directional relationship with stock markets. The author’s empirical evidence shows that asset price fluctuations fundamentally affect future output expectations. [14] claim that an increase in listed companies’ market capitalization and domestic credit and a decline in the interest rate differentials positively affect per capita income in Latin American countries. The authors’ empirical evidence shows that the delayed impact of market capitalization increases GDP per capita. Besides the stock market and economic growth interactions, studies have also focused on contagion effects. For instance, [15] examines the financial contagion of the GFC. The author’s empirical evidence indicates that the Spanish market’s interrelation with the French and British markets displayed a different pattern regarding the post-crisis period. Unlike these two markets, the Spanish market’s connectedness with the German market remained the same at the pre-crisis level. During the pandemic, we have also observed an interest in the financial connectedness of the markets. For example, [16] reveal that the stock markets of China and Saudi Arabia are weakly integrated into the world market. According to the authors, spillovers varied with time and reached the highest levels during the COVID-19 outbreak. [17] report that various spillover patterns exist in high and low volatility regimes and the spillover intensified suddenly in the high volatility regimes during the outbreak. [18] investigate the impact of COVID-19 on China’s economic growth through MIDAS regression analysis. Results indicate a more severe influence from the pandemic than the GFC in conjunction with the superior performance of the MIDAS model against the alternative methods such as Markov regime-switching regression analysis. [19] identify that the volatility spillover between the US and Chinese stock markets was higher during COVID-19 than the pre-COVID-19 period. The authors conclude that the correlation continued during the second wave, although the US administration lifted the general quarantine restrictions of the first wave. The authors’ empirical evidence also reports that the Chinese and US markets have asymmetric effects on the correlation between the two markets. [20] indicate that investors used various financial instruments, including Bitcoin, to find a safer haven. According to the authors, the volatility relationship between Bitcoin and the leading Chinese stock markets evolved significantly during financial stress caused by the COVID-19 outbreak.

[21] find that some regions were able to lessen the economic uncertainty caused by COVID-19 as the crisis evolved. The authors claimed that Google Trends are a proxy for uncertainty, drive returns, and are a trigger for volatility. [22] state that structural problems in the banking system caused the GFC, and classify COVID-19 as a different type of contagion compared to the GFC and war-based crises. The authors’ empirical evidence showed that COVID-19’s effect on geopolitical risk is higher than US economic certainty. [23] show that during the COVID-19 outbreak, financial and non-financial companies’ conditional correlation of stock returns increased considerably by being higher for financial firms. The authors claim that this finding is an indicator of financial firms’ role in financial contagion transmissions. [24] utilized a sample between January 7, 2016 to July 1, 2020 for 14 countries to analyze COVID-19’s effects on the stock markets. The authors state that bivariate systematic risk spillover exists between national and global stock markets. According to the authors, bear markets in European and North American developed markets lead to a spillover in the global markets.

[25] report that global exposure through foreign assets, foreign sales, exports, and imports negatively affects abnormal returns in the short run. In the long run, however, the effect reverses. The authors mention that internationalization makes multinational companies more resilient to COVID-19’s economic impact. [26] points out a financial contagion caused by the COVID-19 outbreak equally affected both emerging and developed markets, and that evaluating the extent of the effects caused by financial contagion is very important for finding alternative causes that deepen the financial crisis. According to [27], oil price is a systematic risk proxy, and helps to capture global growth forecasts in emerging frontier stock markets. [28] examines the effect of the pandemic on currency markets and states that the shockwave effect of the pandemic is about eight times greater than the one caused by the GFC. Additionally, according to the author, emerging countries’ currencies, the Brazilian real and the Turkish lira, received the largest hit from the outbreak. [29] report that any hedge fund’s attempt to avoid systematic risk exposure does not produce the desired results, regardless of the fund’s performance. [30] assess the impact of new cases and death toll statistics on the volatility of US markets. Results illustrate the significant effect of the pandemic on financial market volatilities. [31] report that USD and equity indices are the primary shock transmitters during the pre-COVID-19 period, and that the bond index is the primary transmitter during the outbreak. The authors also state that connectedness positively affects the USD index and increases with the connectedness level. Using Geographically Weighted Regression, [32] reveal the impact of social distancing on employment in Brazil and identify the effect on different regions of the country. The authors show that Brazil’s Northeastern region was more severely affected than the southern region. [33] build networks using quantiles domain from quantile vector autoregressive model’s generalized forecast error variance decomposition for extreme returns. The authors’ empirical analysis shows that the dominant clusters’ connection becomes tighter, and the remaining clusters are well separated during the pandemic.

Using 14 New Zealand indices between January 1, 2019 to August 25, 2020, [34] show that the New Zealand government’s travel restrictions, lockdown, and stimulus response policies have heterogeneous and positive impacts on industry stock indices. The authors state that mandatory lockdown is the only policy that positively affected aggregate stock returns in three response policies of the New Zealand government. Unlike the studies which examine spillover effects between financial markets, [35] explore the impact of the pandemic on the US service sector. Results suggest the presence of a considerably severe influence on the entertainment and airline industries. On the other hand, the hotel industry displays a gradual deterioration especially from small-market-cap companies. Restaurants, however, seem to be relatively more stable than other industries. [36] report that good and bad volatilities’ asymmetric impact varies with time and becomes substantially intense during the outbreak period. The authors state that bad volatility spillovers dominate good volatility spillovers. [37] show that macroeconomic shocks increase volatility asymmetry. The authors claim that Asian markets are the main cause of more substantial negative spillovers. [38] indicate that increased public governance removes the financial development’s negative effect on economic growth. According to the authors, countries with high investment profile scores benefit from economic growth via the stock market. [39] points out a panic on stock markets during the pandemic and asset managers cannot find enough "safe-assets" to hedge against this market panic. [40] report that developed countries are positively correlated before and during COVID-19, but the relationship’s strength decreases during COVID-19.

## 3. Methodology

### 3.1. DCC-GARCH

[41] introduced a generalized version of [42] constant conditional correlation (CCC) model that allows dynamic conditional correlations. Unlike the CCC, the DCC enables correlations (*R*) to be time-varying. The covariance matrix of *k* assets returns can be written as below

$$H_{t}=D_{t}R_{t}D_{t}$$

where *D*_*t*_ is the *k* × *k* diagonal matrix of time varying standard deviations from univariate GARCH model and *R*_*t*_ is the time-varying correlation matrix. The simplest specification of correlation matrix can be given as follows

$${{[R}_{t}]}_{i,j}=\frac{\sum_{s=1}^{t-1}\lambda^{s}\varepsilon_{i,t-s}\varepsilon_{j,t-s}}{\sqrt{\left(\sum_{s=1}^{t-1}\lambda^{s}\varepsilon_{i,t-s}^{2})(\sum_{s=1}^{t-1}\lambda^{s}\varepsilon_{j,t-s}^{2}\right)}}$$

The DCC model satisfies the following specifications

$$r_{t}|I_{t-1}\sim N(0,D_{t}R_{t}D_{t})$$

$$D_{t}^{2}=diag\{\omega_{i}\}+diag(\kappa_{i})\;ο\;r_{t-1\;}r_{t-1}^{\prime }+diag\;\{\lambda_{i}\}\;ο\;D_{t-1}^{2}$$

$$\varepsilon_{t}=D_{t}^{-1}r_{t}$$

$$Q_{t}=S\;ο\;(ı{ı}^{\prime }-\text{A}-\text{B})+\text{A}\;ο\;\varepsilon_{t-1}\varepsilon_{t-1}^{\prime }+B\;ο\;Q_{t-1}$$

$$R_{t}=diag{\left\{Q_{t}\right\}}^{-1}Q_{t}diag{\left\{Q_{t}\right\}}^{-1}$$

where *ε*_*t*_ is the standardized disturbances of the univariate GARCH models.

### 3.2. Total volatility spillover index

[43] introduce a volatility spillover index that employs forecast error variance decompositions and does not depend on the Cholesky-factor identification of vector autoregressions. The model, unlike their precursor of [44], allows directional volatility spillovers. To overcome variable ordering dependency in variance decompositions, the authors utilize the generalized VAR approach of [45] and [46]. Given that the N variable *p*th order VAR model below is covariance stationary,

$$x_{t}=\sum_{i=1}^{p}\Phi_{i}x_{t-i}+\varepsilon_{t}$$

the variance decompositions enable us to evaluate the fraction of the H-step-ahead error variance in predicting *x*_*i*_ that is due to shocks *x*_*j*_. Let $\theta_{ij}^{g}(H)$ denotes the H-step-ahead forecast error variance decompositions,

$$\theta_{ij}^{g}(H)=\frac{\sigma_{jj}^{-1}\sum_{h=0}^{H-1}{\left(e_{i}^{\prime }A_{h}{\sum e}_{j}\right)}^{2}}{\sum_{h=0}^{H-1}\left(e_{i}^{\prime }A_{h}\sum A^{\prime }e_{i}\right)}$$

where *Σ* is the variance matrix for the error vector *ε*, *σ*_*jj*_ is the standard deviation of the error term in *j*th equation and finally, *e*_*i*_ is the selection vector with 1 as the *i*th element and 0 otherwise. $\sum_{j=1}^{N}\theta_{ij}^{g}(H)$ does not need to be equal 1. Using this framework total volatility spillover index can be written as follows

$$S^{g}(H)=\frac{\sum_{\begin{array}{c} i,j=1\\ i\neq 1 \end{array}}^{N}{\tilde{\theta }}_{ij}^{g}(H)}{\sum_{i,j=1}^{N}{\tilde{\theta }}_{ij}^{g}(H)}\times 100$$

where, $\sum_{j=1}^{N}{\tilde{\theta }}_{ij}^{g}(H)=1$ and $\sum_{i,j=1}^{N}{\tilde{\theta }}_{ij}^{g}(H)=N$

## 4. Empirical analysis

Although almost all economic or financial crises have different dynamics, they may illustrate similarities in their nature and consequences. For instance, as stated by [47] and [48], while sudden shifts in the expectations of market participants and evaporated trust were the critical determinants of the initial financial chaos of the Asian crisis in 1997, the determinants of the Russian crisis in 1998 were associated with a variety of economic conditions, such as large budget deficits and insufficient foreign reserves. However, both ended up with a currency crisis. On the other hand, although the GFC was not a currency crisis, lack of trust among financial institutions played an essential role in converting the financial distress in the housing market to a liquidity crunch in the banking sector, and then to a sovereign debt crisis in European countries. As stated by [49], the Asian Crisis and the GFC have similarities in terms of co-movements of markets due to the integrations; thus, the financial contagion induced by the GFC can be compared with the limited and regional contagious effects of the Asian crisis. Finally, the recent pandemic also has induced global financial turmoil and spillovers in risk and returns. Therefore, although their dynamics were different in inception, the aftermath of both the GFC and COVID-19 pandemic is quite similar and necessitates an econometric investigation to reveal and compare their contagion channels and spillovers. Although some studies investigate the spillovers and the presence of the China-centered contagion, we have seen that either the period examined or the formation of the pairs are insufficient to account for these dynamics and compare the role of China with other countries. For instance, the evidence reported by [26] is based on the pairs formed only with China and does not substitute it with an alternative market as a source of the pandemic. Additionally, [50] point out spillovers from China only towards South Asian countries. Additionally, in their econometric investigations, authors do not accommodate fat tails that might occur due to the structural breaks (see [51] and [52]).

In the empirical section of the study, the influence of the COVID-19 pandemic is examined on various stock market correlations and volatility spillovers. Besides the analysis of the COVID-19 pandemic, evidence is also provided from the GFC and the impact of both crises is evaluated on a comparison basis. To that end, the results are assessed in short-term and long-term intervals before and after the peak of both financial crises. This formation allows the shockwaves of the GFC and COVID-19 pandemic to be captured. The analysis covers the period from June 6, 2005, to October 6, 2020. Empirical investigations are carried out for the following countries’ equity markets: United States (DJI), the United Kingdom (FTSE), Italy (FTMIB), Spain (IBEX), China (SSEC), and Turkey (XU100). In selecting the stock markets, four developed (US, UK, Italy, Spain) and two emerging countries (China, Turkey) are employed. Although the developed economies sample consists of four countries, the US and the UK are assessed in a separate subgroup following the weights of Italy and Spain in MSCI market classification as discussed above. The index data consist of daily observations and is obtained from the Refinitiv Eikon database. Econometric analyses are conducted through various software such as MATLAB, Gauss, R and Ox-Metrics.

**Fig 1:**
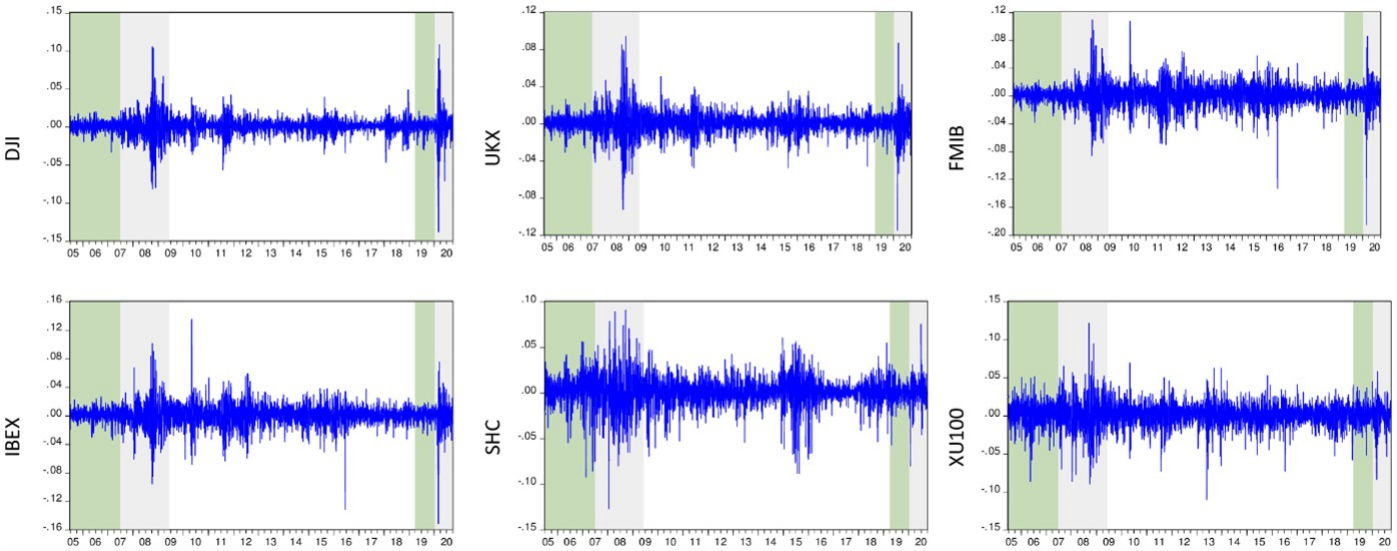
Return series of stock indexes. Green and gray areas demonstrate the pre-crisis and crisis periods, respectively.

Fig 1 illustrates the return series of stock market indexes. To make the behavior of the return series more visible, the crisis periods have been highlighted. The time intervals of the GFC and the COVID-19 pandemic (in gray) are defined as follows: June 19, 2007–May 29, 2009, and January 2, 2020–October 6, 2020, respectively. In identifying the crisis period for the GFC, the timeline of [53] is followed as recommended by [54]. As for the pandemic period, this study references China’s official report to the World Health Organization (WHO) of the first case of the pandemic on December 31, 2019. To obtain robust test statistics, we also allocated an equal number of observations for tranquil periods (in green). Thus, the pre-crisis periods consist of the following time range: July 06, 2005–June 18, 2007, and March 28, 2019–December 31, 2019, respectively. The behavior of the series depicts that both turbulence periods cause severe fluctuations in the return series. Substantial variability in the Chinese stock market returns is also observed in 2015 and 2016. The fluctuation in the Chinese market can be evaluated as price correction following a stock market bubble. The plunge in the market and the steep rise in asset prices did not correlate with the trend in global stock markets in this period. Therefore, the volatility in 2015–2016 seems more related to the internal factors of China, such as margin financing and herd behavior. The patterns of the variables are quite similar in DJI and FTSE, FTMIB and IBEX, and SSEC and XU100 pairs. These clusters are consistent with the global market segmentation of the selected countries.

Table 1 presents the descriptive statistics of the index returns during pre-crisis and crisis days. According to the average returns, all countries display a negative mean during both financial crises except for Turkish and Chinese stock markets seen in the global pandemic. The impact of these crises can also be seen in standard deviations. All return series display a sharp volatility rise during the GFC. While the average standard deviation of variables is 0.0104 in the pre-crisis period, it increases to 0.022 in conjunction with GFC turbulence. A similar case is seen in the COVID-19 pandemic. The average standard deviation rises from 0.0091 to 0.0209 after the crisis. Skewness and kurtosis statistics show that the return series exhibit non-normal distributions in each period. Although the pre-GFC period returns are negatively skewed, they display positive skewness following the crisis except for the Chinese market. However, this change is not observed in the COVID-19 pandemic. After the pandemic, stock returns are still negatively skewed. This phenomenon means that the frequency of the positive returns is still higher than the negative returns. Kurtosis values also demonstrate significant changes, and for each period, returns have heavy tails due to the turmoil in the market. Following the crisis, the tails of the return distributions become thicker except in the case of the Chinese stock markets during the GFC.

**Table 1:** Descriptive statistics.

		DJI	FTSE	FTMIB	IBEX	SSEC	XU100
Pre- GFC	Mean	0.0006	0.0005	0.0006	0.0009	0.0029	0.0011
	Std. Dev.	0.0062	0.0074	0.0079	0.0083	0.0160	0.0166
	Skewness	-0.4139	-0.4267	-0.6248	-0.5422	-1.0651	-0.5227
	Kurtosis	5.2637	4.7679	4.8500	4.9327	8.7853	4.8809
	Jarque-Bera	118.86*	78.84*	101.96*	100.48*	777.57*	94.74*
GFC	Mean	-0.0010	-0.0008	-0.0016	-0.0010	-0.0010	-0.0006
	Std. Dev.	0.0204	0.0201	0.0214	0.0209	0.0251	0.0244
	Skewness	0.1777	0.0776	0.1636	0.1062	-0.2235	0.0431
	Kurtosis	7.2897	6.8373	7.0868	6.9636	5.1581	5.3251
	Jarque-Bera	379.05*	301.74*	343.88*	322.32*	99.37*	110.75*
Pre-COVID-19	Mean	0.0006	0.0002	0.0005	0.0002	0.0000	0.0011
	Std. Dev.	0.0075	0.0072	0.0094	0.0078	0.0103	0.0126
	Skewness	-1.0194	-0.7679	-0.5050	-0.5247	-0.5559	-0.0053
	Kurtosis	5.9253	5.7068	4.0000	3.8095	8.1700	4.8321
	Jarque-Bera	102.24*	77.89*	16.24*	14.13*	224.89*	26.99*
COVID-19	Mean	-0.0001	-0.0012	-0.0010	-0.0017	0.0003	0.0000
	Std. Dev.	0.0261	0.0202	0.0247	0.0230	0.0143	0.0174
	Skewness	-0.7308	-1.0174	-2.5864	-1.5706	-0.5306	-1.2101
	Kurtosis	10.0987	9.9931	21.0248	13.0992	11.7932	8.1253
	Jarque-Bera	422.41*	426.56*	2827.86*	899.54*	630.85*	258.34*

* indicates statistical significance at the 1% level.

In Table 2, the results of the [55] m-break unit root test are presented. The model tests the null hypothesis of a unit root against the alternative hypothesis of an unspecified number of breaks. The critical values are -7.395, -6.717 and -6.417 for the 1%, 5% and 10% significance levels. Results show that the null hypothesis is rejected in each confidence level for all return series. Following the determination of stationary of the variables, the M-ICSS tests are executed to identify the structural breaks in the volatilities of indexes.

**Table 2:** Kapetanios m-break unit root test.

		DJI	FTSE	FTMIB	IBEX	SSEC	XU100
GFC	Test Statistic	-28.1459	-25.8586	-23.7116	-24.8240	-33.9704	-30.8456
	Break Dates	07/17/06	06/14/06	05/18/06	01/18/06	05/15/06	12/20/05
		03/16/07	03/14/07	03/02/07	06/13/06	05/23/07	06/26/06
		08/16/07	08/16/07	07/26/07	08/28/07	10/16/07	01/10/07
		03/10/08	03/17/08	03/24/08	01/23/08	04/18/08	12/31/07
		09/26/08	09/02/08	09/25/08	10/03/08	11/04/08	09/08/08
COVID-19	Test Statistic	-31.2365	-21.6813	-24.5467	-24.3612	-19.7868	-21.6707
	Break Dates	06/03/19	08/06/19	05/31/19	08/15/19	05/24/19	05/24/19
		08/15/19	10/03/19	01/15/20	01/15/20	08/07/19	10/16/19
		01/23/20	01/23/20	03/16/20	03/16/20	01/06/20	01/08/20
		03/23/20	03/23/20	05/15/20	05/15/20	03/05/20	03/23/20
		05/20/20	06/01/20	07/31/20	07/31/20	06/29/20	07/27/20

For determining the potential structural breaks in the volatility of time series, [56] propose a new algorithm (M-ICSS) by modifying the original model (ICSS) of [57]. The authors state that the ICSS model has severe flaws and might cause spurious results in the presence of heteroskedastic conditional variance, or leptokurtic and platykurtic innovations in data. In Table 3, the break dates in the volatility of each series are presented with their rank. The M-ICSS test is conducted for the GFC and COVID-19 pandemic periods separately without splitting data into two-time intervals as before. Since the purpose is to detect the break dates, the series is not divided into pre-crisis and crisis periods. By doing so, the algorithm is also allowed to identify potential breaks that contain the shifts due to the GFC and COVID-19. The highest number of breaks is observed in the volatility of IBEX. Accordingly, the descending order of the breaks for the indexes can be given as follows IBEX, FTSE, SSEC, DJI and FTMIB (same number), and XU100. The dates detected illustrate the success of the algorithm in capturing important events. Except for two emerging economies, Turkey and China, the peak day of the GFC (bankruptcy of Lehman Brothers on September 15, 2008) is nearly approximated by the model. As the center of the crisis, the date of July 11, 2007, in the American stock market is also of note. This date can be linked to the collapse of two Bear Stearns hedge funds, which is accepted as the beginning of the turmoil in the US mortgage market.

**Table 3:** Modified ICSS test results.

	DJI		FTSE		FTMIB		IBEX		SSEC		XU100	
GFC	507	07/11/07	207	05/01/06	513	07/19/07	215	05/11/06	61	09/29/05	640	01/18/08
	804	09/12/08	239	06/15/06	804	09/12/08	636	01/14/08	195	04/12/06	-	-
	860	12/02/08	515	07/23/07	864	12/08/08	651	02/05/08	235	06/09/06	-	-
	-	-	804	09/12/08	-	-	747	06/23/08	372	12/22/06	-	-
	-	-	864	12/08/08	-	-	808	09/18/08	-	-	-	-
	-	-	-	-	-	-	864	12/08/08	-	-	-	-
COVID-19	228	02/21/20	228	02/21/20	228	02/21/20	228	02/21/20	-	-	226	02/19/20
	259	04/06/20	253	03/27/20	308	06/16/20	251	03/25/20	-	-	253	03/27/20
	327	07/14/20	308	06/16/20	-	-	308	06/16/20	-	-	-	-
	362	09/01/20	-	-	-	-	-	-	-	-	-	-

In each variable, the first and second columns indicate the sequence number and date of the breaks, respectively.

The pandemic-related break dates are presented in the second panel of Table 3. Across the variables, except for the Chinese and Turkish stock markets, February 21 is considered as a break date in each index. For the Turkish stock market, this date is closely captured in the model, with a break date of February 19. However, the algorithm cannot detect a break in the volatility of the Chinese stock market. This result can be attributed to its inefficiency or strict governance enforced by the Chinese authorities [58, 59].

To identify the co-movements of stock markets, the dynamic conditional correlations of the variable pairs are computed. To avoid inconsistent standard errors and obtain robust coefficients, the DCC-GARCH methodology of [41], which allows for time-varying correlations, is employed. In the forming of variable pairs, DJ and SSEC variables are used as reference indexes for considering the origin of both crises. As the calculated correlations are time-varying, the data is not split into the aforementioned time intervals and the entire period from June 19, 2007, to October 6, 2020 is used in the analysis. The first and second panels in Table 4 present the DCC-GARCH results for the pairs formed for DJI and SSEC, respectively. [51] states that structural breaks can be the primary reason for non-normality. In addition, [52] report that probability distribution, which accommodates thicker tails, is able to capture the effects of structural breaks. As well, [60] discuss the relationship between structural breaks and return distributions. Considering these studies, since the period examined displays structural breaks, student-*t* distribution is used in modeling time-varying conditional correlations.

**Table 4:** Dynamic conditional correlation GARCH model results.

	DJI_FTMIB	DJI_IBEX	DJI_SSEC	DJI_FTSE	DJI_XU100
*ρ*	0.5132*** (0.0446)	0.5012*** (0.0488)	0.0697*** (0.0168)	0.4682*** (0.0910)	0.2427*** (0.1934)
*α*	0.0102** (0.0041)	0.0080** (0.0040)	0.0040 (0.0048)	0.0073*** (0.0025)	0.0088 (0.0139)
*β*	0.9855*** (0.0067)	0.9881*** (0.0079)	0.9582*** (0.0464)	0.9917*** (0.0032)	0.9870*** (0.0335)
*df*	5.9483*** (0.3382)	5.8838*** (0.3367)	5.1423*** (0.2400)	6.0639*** (0.3552)	5.4847*** (0.2786)
AIC	-12.8592	-13.0048	-12.5405	-13.6029	-12.3868
SC	-12.8429	-12.9885	-12.5242	-13.5866	-12.3705
	SSEC_FTMIB	SSEC_IBEX	SSEC_DJI	SSEC_FTSE	SSEC_XU1g00
*ρ*	0.1219*** (0.0172)	0.1131*** (0.0176)	0.0697*** (0.0168)	0.0719*** (0.1016)	0.0934*** (0.0181)
*α*	0.0042 (0.0051)	0.0080 (0.0056)	0.0040 (0.0048)	0.0021* (0.0011)	0.0115 (0.0074)
*β*	0.9501 (0.0187)	0.9343*** (0.0170)	0.9582*** (0.0464)	0.9975*** (0.0014)	0.9015*** (0.0349)
*df*	5.4369*** (0.2762)	5.3740*** (0.2655)	5.1423*** (0.2400)	5.6153*** (0.2930)	4.9219*** (0.2126)
AIC	-11.6759	-11.8287	-12.5405	-12.3793	-11.4875
SC	-11.6597	-11.8124	-12.5242	-12.3630	-11.4712

*, ** and *** indicate statistical significance at the 10%, 5% and 1% level, respectively.

*ρ*, *α* and *β* denote correlation coefficient, ARCH and GARCH parameters, respectively. df: degrees of freedom in student-*t* distribution. AIC: Akaike information criterion, SC: Schwarz Criterion.

**Fig 2:**
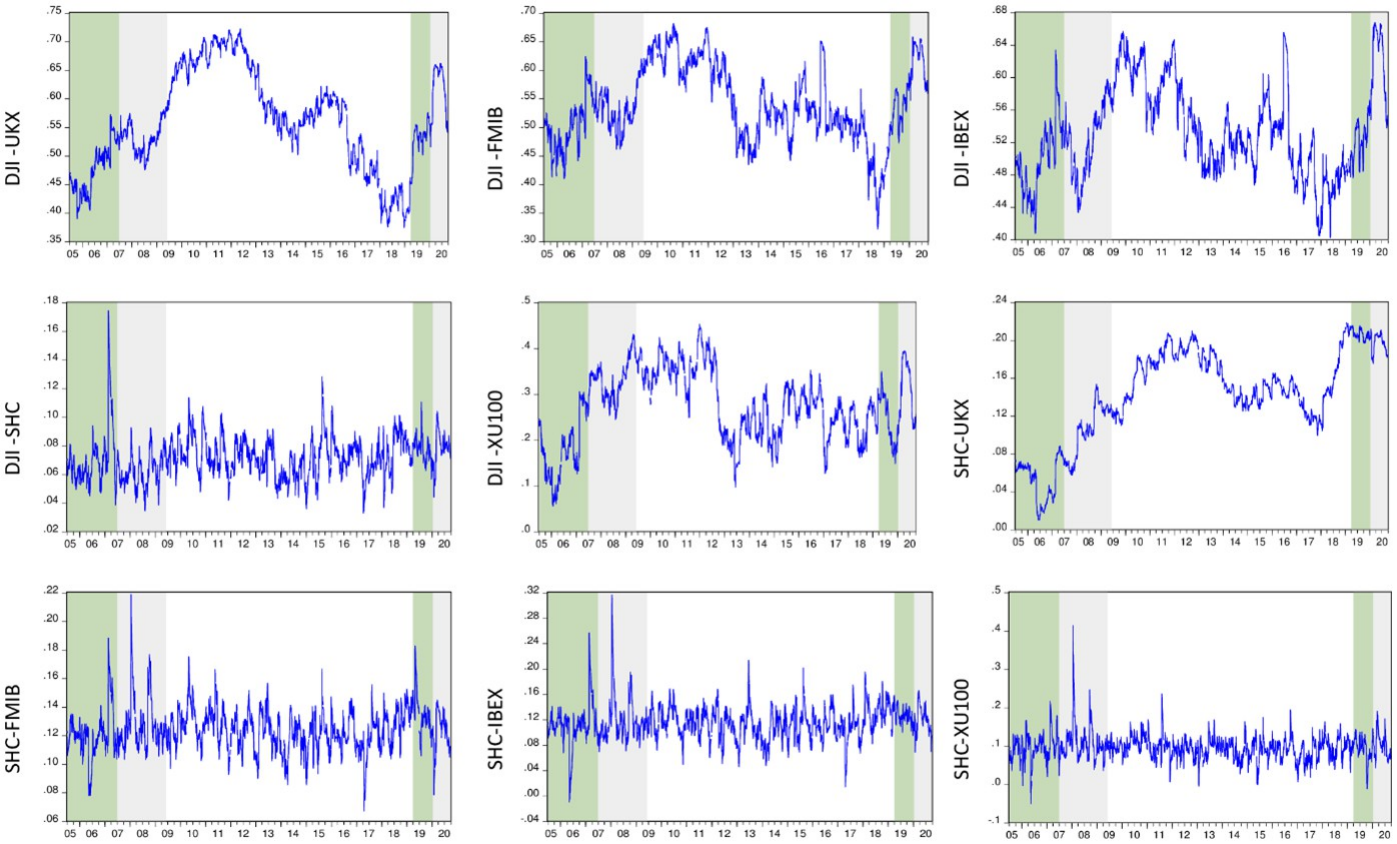
Dynamic conditional correlations of stock markets (DCC-GARCH results). Green and gray areas demonstrate the pre-crisis and crisis periods, respectively.

As the results demonstrate, pairs formed with DJI have higher AIC and SC values than those with SSEC. In Fig 2, we present the time-varying correlations of the variables. The patterns obtained from this analysis can reveal the contagion effect of both crises.

As in the return series, the pre-crisis (green) and crisis (gray) periods are also highlighted in dynamic conditional correlations. Results demonstrate that the co-movements of DJI and other variables are quite similar across the years except for the Chinese stock market. The correlations between DJI and FTSE, DJI and FTMIB, DJI and IBEX, and DJI and XU100 start increasing in 2008 and reach a record high during the European debt crisis. However, this pattern could not be observed in the Chinese stock market. When the SSEC variable is used as a benchmark index to evaluate contagion effects, it is revealed that each pair has a relatively smaller reaction to the developments in international financial markets across the years. Although some jumps are observed on specific dates, there is no upward or downward trend in any correlations except for the FTSE variable. The conditional correlation between SSEC and FTSE demonstrates an upward trend between 2006 and 2013. While it illustrates relatively lower values across 2014–2018, it reached its historical high in 2019. When the behavior of correlations is examined during the COVID-19 pandemic, the contagion effect is most prevalent between developed economies. For example, although the pandemic emerged from China, the propagation of the crisis seems more severe in the following pairs: DJI and FTSE, DJI and FTMIB, DJI and IBEX and DJI and XU100. More interestingly, the Chinese stock market displays a decoupling process from other stock markets during the pandemic’s peak days. While the correlations are soaring in the first quarter of 2020 between DJI and other variables (in the range of 0.40 and 0.65), over the same period, China demonstrates weaker and shorter-term correlations (in the range of 0.10 and 0.15), which later turn into a downward trend.

To measure the severity of the GFC and COVID-19 on stock market co-movements, the changes in the mean value of conditional correlations are also calculated between the pre-crisis and crisis periods. The influence of the crises is examined in two different panels in Table 5. While Panel A covers longer time intervals for the tranquil (July 06, 2005 –June 18, 2007, for GFC and March 28, 2019, and December 31, 2019, for COVID-19) and crisis (June 19, 2007 –May 29, 2009, for GFC and January 02, 2020 –October 06, 2020, for COVID-19) periods of both cases, in Panel B only three-monthly intervals are compared, based on the peak day of both crises. To that end, we utilize the dates detected by the M-ICSS analysis. The reference dates selected for the GFC and COVID-19 pandemic are September 12, 2008, and February 21, 2020, respectively. For both cases, the dates indicate the structural breaks in the volatility of the series. It is evident that the first date corresponds to the collapse of Lehman Brothers (on the next business day, Monday). Similarly, the second date also indicates the plunge in world stock markets during the pandemic. This sharp fall in equity markets proceeded for almost one month, and the variables examined could not beat their previous high till June 10, 2020.

**Table 5:** The change in dynamic conditional correlations in crisis periods.

	Section 1	DJI_FTSE	DJI_FTMIB	DJI_IBEX	DJI_SSEC	DJI_XU100
PANEL A: A Full Period of the Crises	Pre—GFC	0.4742	0.4994	0.4978	0.0706	0.1732
	During GFC	0.5304	0.5308	0.5195	0.0620	0.3283
	Difference	0.0562	0.0314	0.0216	-0.0087	0.1551
	S-W t-test	24.9981***	12.0051***	7.8836***	-8.0268***	43.9801***
	Section 2	SSEC_ DJI	SSEC_FTSE	SSEC_FTMIB	SSEC_IBEX	SSEC_XU100
	Pre-COVID19	0.0778	0.2060	0.1350	0.1266	0.0922
	During COVID19	0.0779	0.1972	0.1229	0.1190	0.1026
	Difference	0.0001	-0.0088	-0.0122	-0.0077	0.0104
	S-W t-test	0.1315	-11.2536***	-8.6699***	-4.4722***	3.3710***
	Section 3	DJI_FTSE	DJI_FTMIB	DJI_IBEX	DJI_SSEC	DJI_XU100
	Pre-COVID19	0.5269	0.5314	0.5224	0.0778	0.2455
	During COVID19	0.6151	0.6141	0.6172	0.0779	0.3073
	Difference	0.0882	0.0827	0.0948	0.0001	0.0618
	S-W t-test	22.2214***	27.1643***	27.1483***	0.1315	10.1444***
	Section 1	DJI_FTSE	DJI_FTMIB	DJI_IBEX	DJI_SSEC	DJI_XU100
PANEL B: Peak Period of the Crises	Pre-BLB	0.4983	0.4932	0.5069	0.0514	0.2822
	Post-BLB	0.5258	0.5251	0.5456	0.0708	0.3250
	Difference	0.0276	0.0319	0.0387	0.0194	0.0428
	S-W t-test	13.3406***	9.5614***	18.1089***	10.4652***	11.7622***
	Section 2	SSEC_ DJI	SSEC_FTSE	SSEC_FTMIB	SSEC_IBEX	SSEC_XU100
	Pre-Feb. 21	0.0666	0.2000	0.1171	0.1095	0.0905
	Post-Feb. 21	0.0850	0.1989	0.1303	0.1334	0.1212
	Difference	0.0184	-0.0010	0.0132	0.0239	0.0306
	S-W t-test	10.465***	19.9538***	9.36939***	9.30473***	3.29777***
	Section 3	DJI_FTSE	DJI_FTMIB	DJI_IBEX	DJI_SSEC	DJI_XU100
	Pre-Feb. 21	0.5550	0.5751	0.5639	0.0666	0.2105
	Post-Feb. 21	0.6451	0.6388	0.6528	0.0850	0.3638
	Difference	0.0901	0.0637	0.0889	0.0184	0.1533
	S-W t-test	13.4112***	9.60813***	18.2088***	10.4652***	11.7622***

S-W and BLB denote Satterthwaite-Welch and the Bankruptcy of Lehman Brothers, respectively. Section 1, 2, and 3 evaluate the contagion effects for two different sources: the US and China. Section 1 considers the US as a source in the GFC, and Section 2 considers China as a source during the pandemic. Finally, Section 3 replaces China and deems the US a source of contagious effects during the global pandemic. Panel A and B examine the same case for long and short-time intervals, respectively.

*** indicates statistical significance at the 1% level.

According to the results in Panel A, the GFC and COVID-19 pandemic caused a different extent of turmoil in global stock markets. The GFC significantly raised the correlations between the USA and other countries’ stock markets except for China (Section 1). However, a similar effect in the case of COVID-19 is not observed when using China as a reference country (Section 2) to form pairs. For instance, while the average change in the GFC is 0.051 in Section 1, it is -0.0036 in Section 2. However, when China is replaced with the USA in the same panel, the average change increases to 0.0655 (Section 3). This change shows that the USA stock market dominates the financial contagion even though its origin was in China. When the analysis period is shortened to three months, as mentioned above, it is revealed that while Section 1 and Section 2 display similar results with their correspondents in Panel A, section 3, which employs the USA as a benchmark country, exhibits greater average changes. The Satterthwaite-Welch t-test statistics are also provided to test the significance of these findings. According to the results, out of thirty test statistics, twenty-seven are statistically significant.

**Fig 3:**
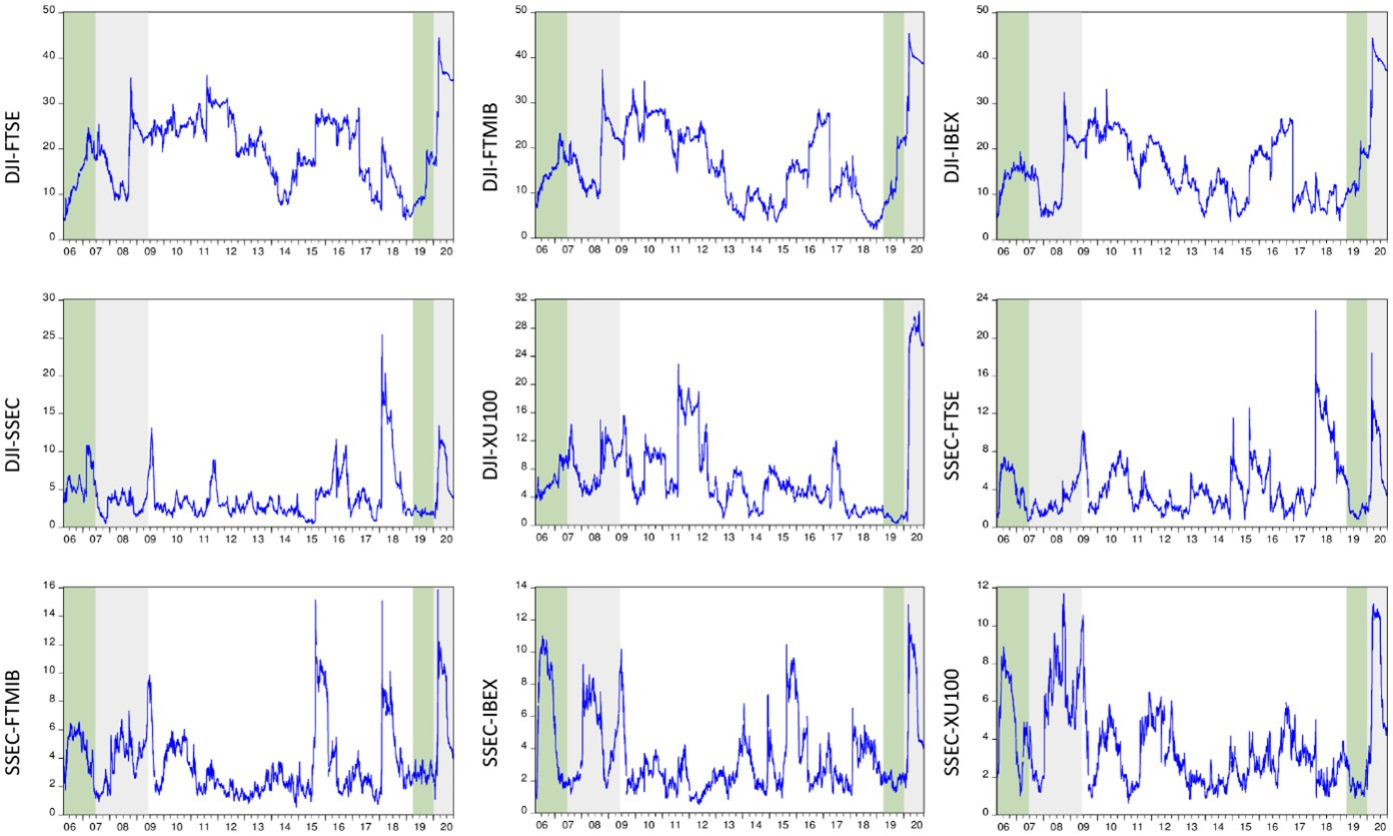
Total volatility spillovers in stock markets (Diebold-Yilmaz method). Green and gray areas demonstrate the pre-crisis and crisis periods, respectively.

The total volatility spillover in each market pair is shown in Fig 3. This analysis is conducted using the methodology of [43]. Unlike [44], which rely on Cholesky-factor identification in vector autoregressions, this model is independent of variable ordering in variance decompositions. This new approach allows for a time-varying analysis of directional volatility spillovers. In executing the analysis, following [43], the rolling-window size is set to 200 days. The VAR lag length and forecast horizon are four days and ten days, respectively.

As seen in Fig 3, time-varying total volatility spillovers spike during each financial crisis. When using the DJI variable as the source of volatility transmissions, the index value presents very sharp rises during the GFC, especially in European stock markets. The extent of stress exposure in the Chinese and Turkish stock markets is relatively low. However, when we move on to the COVID-19 pandemic, it is clear that the level of turmoil shared in the market is considerably higher than that seen in the DJI and FTSE, DJI and FTMIB, DJI and IBEX, and DJI and XU100 pairs. This finding means that the volatility forecast error variance reached its record high in these pairs due to the pandemic. Once again, however, China demonstrates a different image, as seen in the dynamic conditional correlations. According to the results, although the total volatility spillovers display a spike during the pandemic, the extent of the market tension seems to have not reached its previous highs in most cases.

Table 6 presents the changes in total volatility spillovers due to the GFC and COVID-19 in various market pairs. The table follows a similar structure to Table 5. As before, Panel A and B examine two different time intervals. While Panel A compares a longer period for both the GFC and COVID-19 pandemic, Panel B employs three-monthly comparisons around the peaks of both crises, namely the bankruptcy of Lehman Brothers on September 15, 2008, and the plunge in stock markets on February 21, 2020. The M-ICSS analysis justifies all these dates. The significance of changes in average total volatility spillovers in each comparison is also checked through the Satterthwaite-Welch t-test. According to the results, the increase in tension shared in the market is higher during the COVID-19 pandemic, regardless of whether SSEC or DJI is used as a reference index. However, it is evident that if the DJI variable is used as a benchmark in pairs (Section 3) instead of SSEC (Section 2), the average change becomes 3.86 times greater. This result shows that even though the crisis emerged in China and spread worldwide, in its transmission, the US stock market plays a more dominant role than the Chinese stock market. Results in Panel B show that when we shorten the period and focus on the breaks in the failure of Lehman Brothers on September 15, 2008, and the plunge of stock markets on February 21, 2020, the changes in total volatility spillovers are consistent with the changes in conditional correlations. While the crisis emerged in China, the largest volatility spillovers are observed in the pairs that contain the DJI variable. Panel B shows that the COVID-19 pandemic induced a more severe rise in the total market tension than the collapse of Lehman Brothers and that the total volatility spillovers are predominantly led by the US stock markets instead of their Chinese counterparts.

**Table 6:** The change in total volatility spillovers in crisis periods.

	Section 1	DJI_FTSE	DJI_FTMIB	DJI_IBEX	DJI_SSEC	DJI_XU100
PANEL A: A Full Period of the Crises	Pre—GFC	14.04	14.67	13.66	6.02	6.26
	During GFC	17.84	17.56	13.62	3.01	8.22
	Difference	3.80	2.89	-0.05	-3.01	1.96
	S-W t-test	8.7726***	7.8655***	-0.1348	-23.143***	11.809***
	Section 2	SSEC_ DJI	SSEC_FTSE	SSEC_FTMIB	SSEC_IBEX	SSEC_XU100
	Pre-COVID19	1.91	1.98	2.90	2.01	1.66
	During COVID19	6.65	6.83	6.88	6.40	6.90
	Difference	4.74	4.85	3.98	4.39	5.24
	S-W t-test	18.5581***	17.3511***	16.3000***	18.6706***	22.1860***
	Section 3	DJI_FTSE	DJI_FTMIB	DJI_IBEX	DJI_SSEC	DJI_XU100
	Pre-COVID19	11.49	12.79	13.83	1.91	0.96
	During COVID19	32.32	35.82	34.93	6.65	20.70
	Difference	20.83	23.02	21.10	4.74	19.75
	S-W t-test	30.9012***	34.4149***	32.0814***	18.5581***	24.7861***
	Section 1	DJI_FTSE	DJI_FTMIB	DJI_IBEX	DJI_SSEC	DJI_XU100
PANEL B: Peak Period of the Crises	Pre-BLB	10.42	10.72	7.47	3.90	5.88
	Post- BLB	25.54	26.03	22.18	3.30	10.50
	Difference	15.12	15.31	14.71	-0.60	4.62
	S-W t-test	20.7017***	20.2724***	21.1949***	-3.88691***	18.5068***
	Section 2	SSEC_ DJI	SSEC_FTSE	SSEC_FTMIB	SSEC_IBEX	SSEC_XU100
	Pre-Feb. 21	1.82	1.97	3.25	2.19	1.65
	Post-Feb. 21	9.33	9.94	9.58	8.92	8.88
	Difference	7.50	7.97	6.34	6.74	7.23
	S-W t-test	19.5746***	19.4790***	18.5926***	19.1429***	17.9072***
	Section 3	DJI_FTSE	DJI_FTMIB	DJI_IBEX	DJI_SSEC	DJI_XU100
	Pre-Feb. 21	18.56	21.45	19.04	1.82	1.20
	Post-Feb. 21	35.05	37.81	37.63	9.33	20.87
	Difference	16.49	16.36	18.59	7.50	19.67
	S-W t-test	19.5285***	19.5646***	22.3502***	19.5746***	15.7074***

S-W and BLB denote Satterthwaite-Welch and the Bankruptcy of Lehman Brothers, respectively. Section 1, 2, and 3 evaluate the contagion effects for two different sources: the US and China. Section 1 considers the US as a source in the GFC, and Section 2 considers China as a source during the pandemic. Finally, Section 3 replaces China and deems the US a source of contagious effects during the global pandemic. Panel A and B examine the same case for long and short-time intervals, respectively.

*** indicates statistical significance at the 1% level.

This study shows evidence for cross-market linkages of different countries from various development levels. Results differ from the studies that focus on the similar connection of various markets. For example, [26] and [61] report significant contagion and spillovers from China to its trade partners during the pandemic. On the other hand, the results of this study indicate that apart from being the origin of the outbreak, China-based spillovers have a relatively limited impact on other equity markets regarding the transmission of shocks. These findings may indicate that financial connectedness across the markets can be more dominant in the transmission of shocks than real economic linkages. When the role of the US is considered in global markets, it is found that as with the virus itself, the form and severity of the contagion effects and its spillovers are also transformed and aligned with the dominance of the country in global equity markets. The findings of this study can be accounted for by the distorted expectations of investors and the public due to the sentiment caused by conventional and social media. As one of the principles of finance theory, expectations have an impact on the decisions of investors. Therefore, their reflections on asset prices come as no surprise.

In addition to the empirical findings, and considering the early warning indicator feature of equity markets and the dynamic structure of the pandemic, the potential deteriorations in specific economies and sectors must be considered. As known, following the first hit that occurred in China, the world’s manufacturing center and the first stage in the global supply chain, the second shock wave hit global demand. These developments substantially and negatively impacted public and investor expectations, mainly because of disruptions to cash flows. Since cash flows are the essential input for the value of any asset, along with the distorted expectations, equity markets collapsed. Restrictions on socializing and high unemployment have played a substantial role in the declining demand for goods and services. Plausibly, this economic interaction first impacted companies’ cash flows, specifically operating cash flows. This result can be explained in two possible ways. First, a decline in demand has caused a decrease in revenues, and companies with high operating leverage suffered more than their counterparts with low operating leverage. Second, financial turmoil has triggered financial distress and uncollected receivables for particular sectors (such as the service sector) where the economic effects of the outbreak could not be recovered through measures such as vaccination and normalization in social life. Due to these facts, the companies in these sectors may be expected to report lower net income because of decreased revenues, uncovered fixed costs, and increased bad debt expenses in the near future. The Organization for Economic Co-Operation and Development [62] also indicates that increases in non-performing loans (NPL), especially in emerging market economies, will exceed their historical high regardless of the execution of fiscal or monetary policy measures. These findings display the presence of insufficient capital adequacy ratio risk and fragile banking systems in emerging countries. This observation highlights the potential vulnerabilities in these economies due to the possible waves of the pandemic as per the report of OECD. This forecast would force the banks of emerging economies to initiate extra measures against expenses caused by NPLs. Besides our empirical evidence regarding the presence of high spillovers among developed economies, consideration of OECD predictions may provide better fiscal and monetary measures for the emerging market policymakers. All these facts and the nexus between economic and financial indicators suggest that in the near future, besides developed markets, the global economy might suffer from a high level of spillovers and contagious effects between emerging markets originating from banking system exposures. These developments might induce high risk premiums in the equity markets of these countries.

## 5. Conclusion

Since equity markets are considered the barometers of economic activities, their standalone fluctuations, integrations, co-movements, and volatility transmissions are important for all market participants. During economic turmoil, the spread of returns and risks may cause extreme price movements and panic trigger a market crash. Therefore, determining the direction and extent of risk and return spillovers is of utmost importance for authorities and asset managers that need to take corresponding actions against economic downturn and potential portfolio losses. The turmoil during the COVID-19 pandemic shows that equity markets are also open to the shocks of pandemics. In this study, the influence of COVID-19 on six different stock market indexes is examined, namely, those of the DJI (United States), FTSE (United Kingdom), FTMIB (Italy), IBEX (Spain), SSEC (China), and XU100 (Turkey). To better understand the extent of the crisis, the impact of the pandemic is compared with the GFC. To that end, the index returns examined are in the range of May 07, 2005 –October 6, 2020. For both events, we defined pre-crisis and crisis periods. The tranquil period and crisis days are selected as follows for GFC and COVID-19: July 06, 2005 –June 18, 2007, and June 19, 2007 –May 29, 2009; and March 28, 2019 –December 31, 2019, and January 02, 2020 –October 06, 2020, respectively. The M-ICSS analysis successfully captured the inception and peak day (bankruptcy of Lehman Brothers) of the GFC in variables’ volatilities. It also accurately detected the plunge of stock markets on February 21, 2020.

To examine the financial contagion and volatility spillovers during the GFC and COVID-19 pandemic, DCC-GARCH and total volatility spillover analysis of Diebold-Yilmaz is employed. In both analyses, variable pairs are formed that use DJI and SSEC as reference indexes. According to the findings of the DCC-GARCH model, all variable pairs that contain DJI (DJI and FTSE, DJI and FTMIB, DJI and IBEX, and DJI and XU100) present higher contagion effects during the GFC and COVID-19 pandemic. However, the pairs formed with SSEC display very weak correlations. For example, while the DJI variable has a correlation with other stock indexes in the range of 0.40 and 0.65 during the pandemic, its co-movements with SSEC are quite low during the same days (in the range of 0.10 and 0.15). To examine this relationship, the mean dynamic conditional correlation values are also calculated for pre-crisis and crisis periods. Out of 30 pairs, 27 are found to have statistically significant changes in their correlations. These results also indicate that even though China was the origin of the outbreak, the US stock market is the primary source of financial contagion during the COVID-19 pandemic. Results of the total volatility spillover also support this finding. The DJI pairs display an unprecedented rise (up to 40) in total volatility transmissions during the pandemic. However, when the DJI is replaced with the SSEC, it is seen that, while the index value soars with the COVID-19 pandemic, it does not demonstrate a greater risk exposure than its historical high and fluctuates in the range of 10–20. In comparing tranquil and crisis periods, the changes in total volatility spillovers due to the GFC and pandemic reveal that pairs containing SSEC have very low values, though the results spike when we substitute SSEC with DJI.

This study reveals that the center of financial turmoil and its cross-market linkages are independent of the origin of the outbreak. Even though the origin of the pandemic was in China, it is revealed that the US has dominated the global equity markets in terms of contagion effects and volatility spillovers during the GFC and COVID-19 pandemic. Secondly, this study finds that developed economies have a greater extent of transmissions on spillovers and contagious effects. Finally, it appears that equity markets have rapidly priced global expectations. Considering these observations, it can be concluded that although developed countries are relatively more capable of coping with crises, they are prone to receive and transmit a greater extent of shocks in financial markets. The essential factor in this insight might be the role of emerging countries, especially China, in the global supply chain. Thus, the relatively higher stress, enhanced spillovers, and contagious effects among developed markets might be linked to the distortions in forecasted cash flows. The integration of the markets has catalyzed the extent of this financial distress. All things considered, economic actors might utilize these findings to take corresponding actions and mitigate the potential damage. On the other hand, since this evidence obtained from equity markets and these markets can incorporate historical and publicly available information, our findings can be employed by a large segment of society as much as the market professionals and policymakers.
